# Co-producing an intervention to reduce sedentary behaviour in community-dwelling older adults aged ≥ 75 informed by behaviour change theory

**DOI:** 10.1186/s12877-025-05844-6

**Published:** 2025-03-27

**Authors:** Ragy Tadrous, Anne Forster, Amanda Farrin, Peter A. Coventry, Andrew Clegg

**Affiliations:** 1 University of Leeds, Bradford Institute for Health Research, Academic Unit for Ageing and Stroke Research, Temple Bank House, Bradford Royal Infirmary, Duckworth Lane, Bradford, BD9 6RJ UK; 2 Leeds Institute for Clinical Trials Research, University of Leeds, Leeds, UK; 3 Department of Health Sciences, University of York, York, UK

**Keywords:** Sedentary behaviour, Older adults, Frailty, Behaviour change, Co-production

## Abstract

**Background:**

Older adults are the fastest-growing and most sedentary group in society. With sedentary behaviour associated with negative health outcomes, reducing sedentary time may improve overall well-being. Adults aged ≥ 75 years are underrepresented in sedentary behaviour research, and tailored strategies to reduce sedentary time may be warranted. The development of an intervention to reduce sedentary behaviour in adults aged ≥ 75 years using co-production and behaviour change theory is reported.

**Methods:**

Four co-production workshops with community-dwelling older adults aged ≥ 75 years were held between October-December 2022. The intervention development process was informed by the Behaviour Change Wheel (BCW) and Theoretical Domains Framework (TDF). Audio recordings and workshop notes were iteratively analysed, with findings used to inform subsequent workshops.

**Results:**

The co-production group consisted of six community-dwelling older adults aged ≥ 75 years and two researchers. The developed intervention consists of four components (activity monitoring, educational material, group sessions and researcher follow-up), maps to 24 behaviour change techniques and targets barriers to reducing sedentary time. Participants were receptive of the co-production process.

**Conclusions:**

Integrating co-production with the BCW can provide several benefits, with the BCW providing structure to the intervention development process, and co-production increasing the likelihood of the developed intervention being viewed as feasible by older adults. Furthermore, coding intervention components to the BCW may further our understanding of what approaches are successful at influencing behavioural change. Transparent reporting of the intervention development process may benefit researchers developing interventions with older adults. Future research will pilot the co-produced intervention.

**Supplementary Information:**

The online version contains supplementary material available at 10.1186/s12877-025-05844-6.

## Introduction

Sedentary behaviour is described as any waking behaviour characterised by an energy expenditure ≤ 1.5 METs while seated or reclined [1]. Common sedentary behaviours performed by older adults include watching television, reading, doing puzzles, and knitting [2–4]. Older adults are the fastest-growing segment of the world population; and with approximately 67% of older adults spending over 8.5 h per day sedentary [5], they are also the most sedentary group in society [6, 7]. From a 24-h activity cycle perspective [8], it is important to address prolonged sedentary behaviour as older adults can spend 65–80% of their waking day being sedentary [9]. Sedentary behaviour in older adults is associated with several deleterious outcomes, including hypertension [10], hyperglycaemia [11] and obesity [12]. Additionally, sedentary behaviour can reduce social opportunities, roles and relationships and contribute to poorer cognitive function [13]. The physical and social burden of sedentary behaviour in older adults in the United Kingdom accounts will account for £120 million annually by 2030 [14]. The importance of reducing sedentary behaviour in older adults has been highlighted by the World Health Organisation [15].

The population of adults aged ≥ 75 years is considerably underrepresented in sedentary behaviour research, with previous reviews by Chastin et al.[16] and Ramalho et al.[17] only including one randomised controlled trial, and five qualitative studies conducted in community-dwelling adults aged above 75 years, respectively. As such, our understanding of sedentary behaviour, and the effectiveness of sedentary behaviour interventions in this population have been disproportionally informed by a younger subset of older adults.

As individuals age their care needs typically increase, with an Age UK briefing reporting that the percentage of people experiencing difficulty with activities of daily living (ADLs) increases from 15% in those aged 65–69 to 1-in-3 people requiring some level of care and support by age 85 [18]. The importance of social engagement to improve physical and mental wellbeing and promote healthy ageing has been highlighted in a review by Dogra et al.[19]. Social isolation and reduced social support networks are significantly associated with ageing and have been shown to predispose to increased sedentary time [20]. Such barriers may require tailored strategies to target reducing sedentary behaviour. However, reducing sedentary time in this age group can be challenging due to increased frailty [21], balance impairments [22] and cognitive decline [23, 24]. With the population of older adults aged ≥ 75 years expected to double by 2039 [25], developing effective interventions that account for their diverse levels of sedentary behaviour, attitudes, and care requirements [18, 26] is crucial.

The Medical Research Council (MRC) highlights the importance of combining evidence, theory, and stakeholder involvement for developing and evaluating interventions [27]. O’Cathain et al.[28] outline eight approaches to intervention development including 1) Partnership, 2) Target-population centred, 3) Evidence and theory-based, 4) Implementation-based, 5) Efficiency-based 6) Stepped or phased approaches 7) Intervention-specific, and 8) Combination approaches. Frameworks, such as the Theoretical Domains Framework (TDF), COM-B model, and Behavioural Change Wheel (BCW), provide structured approaches for designing interventions. The TDF highlights cognitive, social, and environmental influences on behaviour [29, 30], while the COM-B emphasises capability, opportunity and motivation as key behavioural determinants [31]. The BCW incorporates these frameworks to analyse and guide intervention development through the use of intervention functions, policy categories, and behaviour change techniques (BCTs) [32]. The BCW requires involving the target population in the intervention development process, and may be particularly suitable for older adults as it allows interventions to meet their specific needs, preferences and capabilities [33]. Although MRC guidance advocates combining stakeholder input and theory when developing complex interventions, it lacks specificity on how this might be done in practical contexts [27]. Partnership approaches ensure collaboration and shared decision-making between researchers and end-users and increase the likelihood that developed solutions are acceptable and feasible [34–37].

There is a lack of interventions targeting sedentary behaviour reduction in older adults that integrate partnership and theory- and evidence-based approaches during development. The Frail-LESS intervention by Bailey et al.[38], aimed at reducing sedentary behaviour in older adults with sarcopenia and frailty was developed using the BCW. Similarly, Leask et al.[36] co-created an intervention to reduce sedentary behaviour. Combination approaches recognise the difficulty of facilitating behaviour change and can assist the integration of evidence and theory into participatory action research designs [33]. This study aimed to co-produce an intervention to reduce sedentary behaviour in community-dwelling older adults aged ≥ 75 years, guided by the BCW. The objectives were as follows:

i. To conduct co-production workshops with stakeholders to design an intervention to reduce sedentary behaviour in older adults, informed by the BCW and findings from a previous mixed-method review.
ii. To develop a logic model outlining key intervention elements including mechanisms of action, mediators, and expected outcomes.
iii. To develop intervention materials that incorporate behaviour change strategies to reduce sedentary behaviour in community-dwelling older adults aged ≥ 75 years.

## Methods

With reference to the methodology described by Hall et al. [39], we conducted four semi-structured focus groups between October and December 2022 at the Bradford Institute for Health Research with adults aged ≥ 75 years to co-produce an intervention to reduce sedentary behaviour [40]. The co-production process, grounded in the BCW, is particularly suited for the target population, as it can enhance the feasibility of the developed intervention, with the BCW providing a structured framework to identify key intervention components, mechanisms of action and BCTs [41]. Focus groups are recommended to help mitigate potential power differences between participants and researchers, which is vital for shared decision-making necessary for co-production [42]. The first two focus groups provide a qualitative profile of sedentary behaviour that will be reported elsewhere [43]. Ethical approval was obtained from the University of Leeds School of Medicine Research Ethics Committee (project number MREC-21–052).

### Recruitment

Participants were recruited from the Community Ageing Research 75 + Study (CARE75 +) cohort. The CARE75 + cohort is a longitudinal cohort study of community-dwelling older adults. The cohort consists of over 1000 older adults aged 75 or above, of which participants optionally consent to be contacted about future research. The CARE75 + study is led by the Academic Unit for Ageing and Stroke Research (ASR), University of Leeds, based at the BIHR, Bradford Teaching Hospitals NHS Foundation Trust. CARE75 + is funded by the National Institute for Health and Care Research, Yorkshire and Humber Applied Research Collaborations NIHR200166 [44, 45].

The recruitment process was conducted over a three-week period. The lead author (RT) collaborated with the CARE75 + cohort manager to identify eligible participants. Potential participants were purposively sampled to ensure a diverse range of sedentary time, frailty, and living arrangements were recruited. Identified participants were sent an advertisement and a participant information sheet. Following seven days, they were contacted by phone, provided with additional details about the study, and were able to ask questions. Those who expressed interest in participating provided verbal consent over the telephone, with written informed consent obtained at the start of the first group meeting. Demographic data information was collected from participants, with frailty classification, measured by the Electronic Frailty Index (eFI) obtained from the CARE75 + cohort, and sedentary time assessed through the Measure of Older Adults' Sedentary Time (MOST) questionnaire at study onset [46]. Experts recommend focus groups of 4–12 participants, ideally 5–10, to encourage discussion without inhibiting input [47, 48]. Over-recruitment is advised to offset cancellations [47]. Thus, we aimed to recruit 5–7 older adults.

### Eligibility criteria

The inclusion criteria were as follows:

- Aged 75 or older
- Residing in the community (not in residential or nursing homes)
- Located within a reasonable distance of the Bradford Institute for Health Research
- Able to stand and walk without assistance
- Willing and able to give written informed consent and proficient in English
- Classified as having mild (0.13–0.24), moderate (0.25–0.36) or severe frailty (> 0.36) according to the eFI.
- Available to attend at least three of four scheduled group sessions

### Principles of co-production

The workshops presented were informed by the principles of co-production, a collaborative process where key stakeholders share authority to design and implement interventions relevant to the target population [37, 49]. This process involves jointly setting the research agenda, devising and executing the research methodology, and analysing, communicating, and applying the research outcomes [50]. This approach followed the framework by Hawkins et al. [51], which included three stages:

1. **Evidence Review and Stakeholder Consultation:** This involves gathering a wide range of perspectives on the target behaviour. These perspectives were gained from a mixed-method review [52].
2. **Co-production:** The co-production group, comprising researchers and older adults, iteratively co-developed the intervention materials through a series of structured workshops. Each session built on the previous one, incorporating participant feedback to refine the intervention components. For example, sedentary activities identified in earlier workshops shaped the development of prompts to reduce sedentary time in the educational booklet.
3. **Prototyping:** Throughout the co-production process, group members tested the various intervention components and provided preliminary insights to refine the intervention components. The acceptability of the developed intervention was also explored through a feasibility study [53].

### Workshop format

The workshops followed an iterative structure, adapting to participant discussions while maintaining alignment with the BCW [51]. Between three to six semi-structured focus groups can sufficiently capture 90% of themes in homogenous study populations [54]. Each two-hour session included three main components:

1. **Introduction and information sharing:** Essential information relevant to the current session and an overview of previous sessions were provided during the meetings' first 15–20 min.
2. **Workshop activities:** The group was divided into smaller subgroups to complete one to two 30–40 min workshop activities that aligned the BCW stages (Table 1). Technical language was minimised for accessibility, using terms like ‘solutions’ or ‘problems’ instead of ‘intervention components’ and ‘behavioural diagnosis’.
3. **Evaluating the workshops:** At the end, subgroups reconvened to reflect on and evaluate the workshops. Feedback was used to enhance participant experience in future workshops. Additional evaluations included researcher reflections and evaluation forms. Participants evaluated the co-production process against the following Co:Create Co-production matrix domains [55]: holistic, resourced, transparent, inclusive, iterative, positive, equal, and sustainable (Supplemental).

**Table 1:** Overview of Workshop Content and Theory

Workshop Title			Workshop Topics			Co-production Activities		Integration of Evidence		Links to BCW		Post-workshop Activities	
1	**Introduction to Sedentary Behaviour**		- Introduction to co-production group and rules of participation - Introduction to sedentary behaviour - Defining and differentiating sedentary behaviour from physical inactivity			- Explore understanding of sedentary behaviour - List activities performed in sitting and standing - Attitudes towards reducing sedentary behaviour		Presentation of evidence from a mixed-method systematic review on reducing sedentary behaviour in community-dwelling older adults		**Stage 1: (Steps 1–3)** - Define the problem - Select the target behaviour - Specify the target behaviour		- Map activities performed in sitting and standing to Ecological model of sedentary behaviour	
2	**Barriers and Facilitators to Reducing Sedentary Behaviour**		- Specification of the target behaviour (who, when, where) - Outlining the barriers and facilitators present to reducing sedentary behaviour in this population			- Subgroup discussions about internal and external barriers and facilitators to reducing sedentary behaviour		Supplementing identified barriers and facilitators with findings from a mixed-method review for discussion		**Stage 1: (Steps 3 & 4)** - Specifying target behaviour - Identifying what needs to change		- Specification target behaviour summarised - Chart barriers and facilitators to COM-B - Behavioural diagnosis	
3	**How Can We Reduce Sedentary Behaviour?**		- Develop solutions to barriers identified in workshop 2 to reduce sedentary behaviour - Prioritisation and appraisal of identified solutions			- Generating solutions based on barriers identified - Tailoring current solutions to better suit this subset of older adults		Presentation on current strategies to reduce sedentary behaviour		**Stages 2 & 3: (Steps 5, 7 & 8)** - Identify intervention functions - Identify delivery modes - Identify behaviour change techniques		- Charted identified solutions to intervention functions and delivery methods - Coded solutions to the BCT	
4	**Reviewing the Intervention**		- Present intervention prototype - Feedback on revisions to the solutions from workshop 3 - Review delivery modalities - Finalise intervention			- Feedback intervention components revisions - Evaluation of co-production process and feedback		Recap of current strategies to reduce sedentary behaviour		**Stages 2 & 3: (Steps 5, 7 & 8)** - Identify intervention functions - Identify delivery modes - Identify behaviour change techniques		- Summary of the co-production process feedback	

### Data collection and analysis

Qualitative data were collected through a combination of audio recordings, worksheets, and field notes. The process of data collection and analysis was iterative, with collected data analysed and used to inform the content of later workshops. For example, in the first workshop, participants identified activities they performed while sitting, which shaped the discussion in the second workshop, where barriers and facilitators to reducing these behaviours were explored. These identified barriers then guided the third workshop, where participants developed strategies to address them. In the final workshop, participants provided feedback on the prototype intervention to ensure it aligned with their perspectives. This included deciding the content and frequency of some components (educational material, group sessions and follow-up phone calls), and the preferred mode of other components. (smartwatch vs self-monitoring).

All workshops were audio-recorded and transcribed verbatim by the lead author (RT). Audio recordings were used to accurately capture the content of the discussion, and inform the thematic analysis. Worksheets provided an additional layer of data, capturing points not explicitly verbalised by participants, for example, activities performed in sitting that were not read aloud. A co-facilitator (SK/SAH) took detailed field notes during each workshop, which were cross-referenced with the transcripts and the worksheets. To ensure the accuracy of the field notes, a verbal summary of the main discussions was presented at the end of each workshop, allowing participants to verify and clarify key points. Field notes also helped guide the data analysis as they highlighted key discussions and their timestamps. Following each session, the lead author and co-facilitator debriefed to discuss the key takeaways from the session. Summaries of the previous workshops were shared at subsequent workshops to ensure continuity and validate findings, and resolve any discrepancies between data sources.

All data from the workshops were coded using NVivo 11 by the lead author (R.T.), with regular input from other members of the research team. An inductive and deductive thematic analysis was conducted using NVivo guided by the methodology described by Braun and Clarke [56]. The inductive analysis explored the participants’ experiences with sedentary behaviour and will be reported elsewhere [43], whereas the deductive analysis was used to chart data according to predefined themes as part of the BCW framework.

### Research team and reflexivity

The lead author, a male researcher of Egyptian Irish descent with a background in Physiotherapy, has significant experience in qualitative research. He received specialised training in qualitative and participatory research methods and facilitated the co-production work. The research team also included four supervisors, from diverse academic backgrounds, with extensive research experience with older adults, intervention development, health psychology and behaviour change. The author group is gender balanced and consisted of junior and senior researchers from various disciplines, with some members belonging to marginalised groups. There were no prior relationships between any member of the research team and the focus group participants before the study began. Participants were informed that this study was part of the lead authors’ doctoral research, and the findings would contribute to the development of an intervention to reduce sedentary behaviour among community-dwelling older adults.

## Results

### Participant characteristics

The co-production group consisted of six older adults and two researchers (RT and SAH/SK). A total of 23 older adults met the eligibility criteria. The most frequently cited reasons for refusing to participate included a lack of interest (*n* = 9), other commitments (*n* = 5), and transport difficulties (*n* = 4). The demographic characteristics of the recruited group members are provided in Table 2. Participants had an average age of 83, five members were male (80%), and participants’ sedentary time ranged from 4 to 13 h per day.

**Table 2:** Characteristics of Recruited Participants

Participants	Gender	Age	Falls within last 12 months	Sedentary hours (weekday)	Sedentary hours (weekend)	Frailty Classification	Ethnicity
1	F	83	2	4	6	Mild	White British
2	M	84	2	8	8	Severe	White British
3	M	82	2	9	10	Moderate	White British
4	M	82	2	10	10	Severe	White British
5	M	84	2	13	13	Moderate	White British
6	M	83	2	13	13	Mild	White British
Average		83	2	9.5	10		
SD		0.89	0	3.4	2.8		

### Workshop attendance, and evaluation

One member could not attend the final workshop due to illness, and the remaining participants attended every session. No participants withdrew from the study. Members of the group evaluated the degree to which the co-production process against the Co:Create Co-production Matrix. Responses ranged from ‘agree’ to ‘strongly agree’ for each domain, and the most well-received domains were ‘Transparent’, ‘Iterative’ and ‘Equality’. Positive feedback from participants included exerts such as “The process couldn’t have been made easier” | P1-83F.

### Intervention development guided by the behaviour change wheel

All aspects of the intervention development process were performed collaboratively, but a deductive thematic analysis was performed with qualitative data being classified according to pre-defined categories, theories or techniques as part of the BCW process for each workshop (Table 1).

### BCW Stage 1: Understanding of the behaviour

To understand a behaviour, it is necessary to define it in behavioural terms by identifying the target population engaged in the behaviour and the behaviour itself [57]. Following this, one must identify the behaviour(s) that need to be addressed to solve the problem, the locations where the behaviour is carried out, and the population involved [57]. This stage of the BCW was informed by a preceding mixed-method review. A behavioural diagnosis was also undertaken in the second workshop.

#### Step 1: Define the behaviour in behavioural terms

This study defined the problem as reducing prolonged sedentary behaviour in community-dwelling older adults. The study focus was conveyed to participants during recruitment and reiterated during the first workshop.

#### Step 2: Selecting the target behaviour

This step involves creating comprehensive lists of all other behaviours that could impact the target behavioural issue. This can be systematically minimised by evaluating the potential influence of each of these behaviours. For this research project, behaviours such as physical inactivity, sedentary behaviour, and sitting time were considered.

#### Step 3: Specifying the target behaviour

Once a target behaviour is chosen, it must be clearly defined with a detailed description of the behaviour and identification. The co-production group chose their preferred terminology during the first workshop (i.e. reduce time spent sitting and lying down). The target behaviour was further specified as reducing excessive sedentary behaviour in community-dwelling older adults. The behaviour is specified according to the criteria described by the BCW in Table 3.

**Table 3:** Specification of Behaviour according to the Behaviour Change Wheel

Considerations		Specification of Target Behaviour
1	Who is responsible for performing it?	Community-dwelling older adults
2	What adjustments are necessary to attain the desired change?	Several intervention components (including goal setting, planning, feedback and monitoring, social support, education, instruction on how to perform behaviour, prompting, and receiving information from credible sources) were positively received by older adults
3	At what time will they execute it	During waking hours
4	In what location will they perform it?	The behaviour will be targeted in the home, during voluntary work, during transport, and during leisure time
5	How frequently will they engage in it?	Daily
6	With whom will they collaborate?	Social support and information from credible sources are valued. Collaboration with other older adults, friends, family or healthcare professionals/carers considered

#### Step 4: Identify what needs to change

The last step of Stage 1 involves identifying necessary changes in the individual and/or surroundings to achieve the desired behavioural change. Intervention developers should conduct a behavioural analysis to identify necessary changes by understanding the target behaviour within its context. A ‘behavioural diagnosis’ was undertaken in the second workshop, identifying barriers and facilitators to reducing sedentary behaviour. Barriers and facilitators were generated by the group members and supplemented with findings from an earlier review.

### BCW Stage 2: Identify intervention options

The COM-B and TDF analyses of the target behaviour were used to identify relevant intervention functions and the policy categories to support their delivery, which were then graded using the APEASE criteria as recommended by the BCW process.

#### Step 5: Identify intervention functions

The COM-B model determines the necessary changes to achieve a desired behaviour and what should be targeted in an intervention. The BCW identifies intervention functions and supporting policies that are likely to be effective in causing change. Specific intervention functions are likely to be effective in bringing about the desired change in the target behaviour for each identified COM-B component. The intervention components identified from the final co-production workshop were coded to seven intervention functions as outlined by the BCW (Table 4). They were as follows: education, enablement, environmental restructuring, incentivisation, modelling, persuasion, and training.

**Table 4:** Theoretical Domains Framework and Behaviour Change Wheel Description of Intervention Components

COM-B Construct	COM-B Domain	TDF Domain	Necessary actions for target behaviour to occur	Participant Quotes	Intervention Function	Relevant BCT	Intervention Component
**Capability**	Psychological	Knowledge	Evidence on why they should reduce their sitting time	“So I'd like to know how they come to the solution that is detrimental to anybody to sit?” **P4-82 M-S**	Education	5.1 Information about health consequences 5.3 Information about social and environmental consequences 9.1 Credible source	Educational booklet Group sessions
			Education about health benefits of reducing sedentary time	“If you could prove to me that it is beneficial?” **P4-82 M-S**			
			Education regarding negative consequences of sedentary time	“So that must indicate that the longer you sit down that the less beneficial it is for your body?” **P3-82 M-Mod**			
			Education regarding social consequences associated with sedentary time	“I do know people that do just sit and sort of look out the window because they don’t know what to do, they have no hobbies” **P1-83F-M**			
			Education regarding strategies to reduce sitting time	“If we essentially sit for an hour less each day? What do you do during that hour?” **P2-84 M-S**			
			Difference between sedentary behaviour and physical inactivity	“I used to think that if I’m active during the day…that I’m doing well and to sit in the evening would be fine…but I’m now reconsidering that.” **P1-83F-M**			
		Memory, Attention, Decision Processes	Increase awareness of sedentary time	“I didn't really think about the amount of time I spent sitting until was asked about it” **P6-83 M-M**	Enablement Environmental restructuring	7.1 Prompts/cues 12.1 Restructuring the physical environment 12.5 Adding objects to the environment	Activity monitoring Prompts
		Behavioural Regulation	Develop strategies for reducing habitual sedentary behaviour and self-monitoring sitting time	(talking about education/effects of group sessions) “I think in the evening it has. Yeah. I know I'm not doing as much moving in an evening. I know, so I definitely am thinking about it. Definitely.” **P1-83F-M**	Education Enablement	1.4 Action planning 2.2 Feedback on behaviour 2.3 Self-monitoring of behaviour 7.1 Prompts/cues 12.5 Adding objects to the environment	Activity monitoring Educational booklet Researcher follow-up Prompts
	Physical	Skills	Targeting daily sedentary behaviours	“I don't want it to be tied to certain days or certain times, I want something that works around me” **P4-82 M-S**	Training	4.1 Instruction on how to perform the behaviour	Educational booklet Group Sessions
**Opportunity**	Social	Social Influences	Social support from a group	“(**referring to group sessions**) We are created to be with each other. We all need people. In particular, loneliness in old age, it meets a need.” **P6-83 M-M** “I think it's interesting to listen to other people's views. Yes. That you differ from your own or make you think down the different avenues” **P3-82 M-Mod**	Modelling Enablement	3.1 Social support (unspecified) 6.2 Social comparison 8.1 Behavioural practice/rehearsal 12.2 Restructuring the social environment	Activity monitoring Group sessions Prompts Researcher follow-up
			Social support from healthcare professionals/researchers	“If you're going to address the thing at all, you've got to answer it and so on, on a one to one basis, particularly on the phone, I think, yeah, it becomes relatively easy to deal with” **P3-82 M-Mod**			
			Combination of group sessions and researcher follow up	“But it occurs to me that one necessarily goes with the other because you want to know how you're progressing. And I think the two go together.” **P3-82 M-Mod** “Of course, we're different as individuals, as a group.” **P6-83 M-M**			
	Physical	Environmental Context and Resources	Technological prompts to reduce sedentary behaviour	(***talking about experience with activity monitors***) “But the more information you can get the better. What I liked as I see you probably inferred was that I discovered I was more active than I thought, I was slightly less ashamed of my sedentary life.” **P6-83 M-M** “It can simulate your thoughts that you should be getting up on a walk” **P3-82 M-Mod** *(talking about activity monitors)* “And if it were telling me to stand up, I would do” **P1-83F-M**	Enablement Environmental restructuring	7.1 Prompts/cues 12.1 Restructuring the physical environment 12.5 Adding objects to the environment	Activity monitoring
			Environmental cues to reduce sedentary behaviour	“One of the tips I have started using because I use public transport every day, more or less. So when I get to the interchange, sometimes I've caught up an hour, whatever. So I'm not sitting down and I'm standing” **P1-83F-M**			
**Motivation**	Reflective	Beliefs about Capabilities	Strong belief about ability to reduce sedentary behaviour	“I will overcome that yeah, if it's anything to overcome, yeah, I don't know. It seems a small thing.” | **P1-83F-M**	Education Persuasion	1.4 Action planning 15.1 Verbal persuasion about capability	Group sessions Researcher follow-up
		Beliefs about Consequences	Education about health benefits of reducing sedentary time	(*Talking about reducing sedentary behaviour)* “…And in what way would that be beneficial?” **P4-82 M-S**	Education	5.1 Information about health consequences 5.3 Information about social and environmental consequences 9.1 Credible source	Group sessions Educational booklet Researcher follow-up
			Education regarding negative health consequences associated with sedentary time	“So I'd like to know how they come to the solution that is detrimental to anybody to sit?” | **P4-82 M-S**			
		Intention	Develop goals or targets to encourage reduction of sedentary behaviour	“Maybe like maybe somebody checking in or somebody following up and working towards a goal, stuff like that.” **P2-84 M-S**	Education Persuasion	1.1 Goal setting 1.4 Action Planning	Researcher follow-up
		Goal	Develop goals or targets to encourage reduction of sedentary behaviour		Incentivisation	1.1 Goal setting (behaviour) 1.2 Problem solving 1.4 Action planning 10.5 Social incentive	Researcher follow-up
	Automatic	Reinforcement	Develop goals or targets to encourage reduction of sedentary behaviour		Persuasion	1.1 Goal setting (behaviour) 1.2 Problem solving 1.4 Action planning 8.2 Behaviour substitution 8.3 Habit formation 8.4 Habit reversal	Activity monitoring Prompts to reduce sedentary time Researcher follow-up
		Emotion	Discuss influence of sedentary behaviour on emotional wellbeing and mood	“And I think mentally also, to just sit. I wouldn’t want to sit a long time. Just sitting.” **P1-83F-M** “I think I'm a little bit more melancholic than you, there are certain times when it really is very tempting, to put your head in a pillow” **P6-83 M-M**	Education Persuasion	2.4 Self-monitoring of outcome(s) of behaviour 5.6 Information about emotional consequences	Group Sessions Researcher follow-up

*KeyM* Mild Frailty, *Mod* Moderate Frailty, *S* Severe Frailty

#### Step 6: Identify policy categories

Group members decided that intervention would be delivered at an individual level and no relevant policy categories were identified. However, the intervention will be piloted, and relevant policies may be identified from participant feedback.

### BCW Stage 3: Identify content and implementation options

After determining the policy categories and intervention functions, the subsequent phase entails identifying particular behaviour change techniques and the mode/s of delivery that are most practically viable within the local context.

#### Step 7: Identify Behaviour change techniques

Intervention components were coded to the most appropriate BCT(s). For example, the education intervention function was deemed most appropriate to address barriers relating to the ‘knowledge’ domain and ‘psychological capability’ component of the TDF and COM-B, respectively, and ‘information about health consequences’ and ‘information about social and environmental consequences’ were deemed the most appropriate BCTs. A total of 23 BCTs were identified (Table 3).

#### Step 8: Identify delivery modalities

Delivery modalities were discussed during the third workshop and refined in the final workshop. Except for group-based sessions, the remaining intervention components would be delivered at an individual level and not require face-to-face contact.

### Habit Formation and intervention duration

As recommended by Lally and Gardner’s habit-formation framework [58], the provisional intervention was designed to: i) Increase motivation to translate the intention of replacing sedentary behaviour into light-intensity physical activity; ii) Support the development of automaticity of reducing sedentary behaviour. iii) Promote continued repetition of the desired behaviour in the presence of the same contextual cues. Furthermore, with automaticity reported to plateau after 66 days, an intervention length of nine weeks was chosen to support ingraining this behaviour change [59].

### Co-produced intervention

**Fig. 1:**
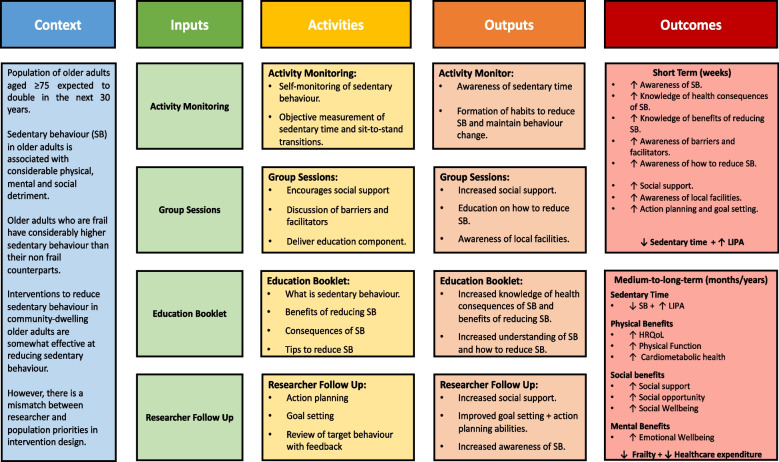
Logic Model of Co-Produced Intervention

The four intervention components were charted to the BCW and TDF in Table 4 and described below. A logic model of the intervention was developed (Fig. 1).

**Activity Monitoring:** Members felt that monitoring their sedentary behaviour was important to increase their awareness of their sedentary time. Some members preferred smartwatches to monitor their sedentary behaviour, which can measure sedentary time and notify them of prolonged sedentary bouts. Others valued self-monitoring through diaries, which they commonly use in daily life. As such, both measures were incorporated in the intervention.

**Educational Material:** Members requested educational material to help them interrupt their sedentary behaviour. The desired content included defining sedentary behaviour, health consequences, health benefits of reducing sedentary time, and practical advice on reducing sedentary time. Prompts to reduce their sedentary behaviour were tailored according to their sedentary activities identified from the first workshop and wider literature. An educational pamphlet was created from the second workshop discussions, presented in Workshop 3, revised based on feedback, and a second prototype was shared in the final workshop (Supplemental).

**Group Sessions:** Members expressed a strong preference for three-to-four group sessions which would serve two primary purposes. Firstly, sessions would include an educational component delivered by a credible source (e.g. a healthcare professional). Secondly, group sessions would provide opportunities to meet other older adults, share advice and develop support networks.

**Follow-Up Calls:** Members felt that follow-up phone calls during the weeks between group sessions would be helpful. These follow-ups would allow them to revisit and progress their goals accordingly and provide opportunities to overcome intervention-related difficulties with assistance. Follow-ups could also be used to document tangible benefits experienced during the intervention, which may promote adherence following cessation.

## Discussion

This study details the methodology for developing a complex intervention to reduce sedentary behaviour in community-dwelling older adults. To our knowledge, this is the second co-produced intervention designed to reduce sedentary behaviour in older adults [36] and the first to focus on adults aged ≥ 75 years. The intervention has since been feasibility-tested and refined, as MRC guidance recommends for developing complex interventions [27].

### Experience with Co-production

Co-production can be a valuable participatory research method that can be used to inform the development of an intervention [50]. A similar approach was used by Leask et al.[36] to co-create an intervention to reduce sedentary time in older adults, and by Giné-Garriga et al.[60] to co-create strategies to reduce sedentary time in care home residents. By involving older adults as shared decision-makers, co-production aims to increase the likelihood that developed interventions are feasible, acceptable and appropriate to the target population [34–36]. This approach builds trust with participants by emphasising their expertise and abilities to make significant contributions. Furthermore, the small size and constant nature of the co-production group helped members feel comfortable contributing to conversations and having constructive debates. The iterative nature of the development process enabled group members to see how their views were actively shaping the intervention which can promote agency, and an important predictor of involvement in and success with co-production [49]. This may reflect the positive feedback received and the high retention of participants during this study.

Another well-received aspect of co-production was the social interactions the group setting provided. Members made direct reference to social benefits when choosing to include a group component in the developed intervention. The social enrichment provided by group sessions may help combat the social isolation that is increasingly encountered in older adults, which can negatively impact physical and emotional well-being in this population [61]. Doing so may also increase older adults’ social support networks, which can decrease in older adulthood following the bereavement of friends and family [62]. However, this group element may exclude certain subsections of older adults from participating in the intervention, as common barriers to attending group sessions, such as poor public transport and physical health problems, can also promote sedentary behaviour [63, 64]. Strategies to overcome this barrier will be explored and may include similar remote social support options described in the Frail-LESS trial [38]. Through co-production, group members navigated the steps outlined in the Creating Domain of the taxonomy of approaches for developing health interventions [28]. Between the third and final workshop, members of the co-production group were able to trial components of the intervention. They were able to give feedback on certain aspects of the intervention including the researcher follow-up, group sessions, and educational booklet, and some members had experience with using devices with a sedentary reminder function. In doing so, feedback from the target population was obtained about the proposed intervention during its development and provided valuable information prior to feasibility-testing.

### Experience with the behaviour change wheel

The BCW provided clear stages to structure the co-production workshops, making the process reproducible and addressing limitations identified in the previous co-created intervention described by Leask et al.[36]. The behavioural diagnosis conducted in the first two workshops served as a useful activity that introduced findings from the literature in an accessible manner to upskill group members about the topic. Furthermore, it provided a sense of agency as it allowed group members to prioritise the key barriers and facilitators to reducing sedentary behaviour in this population from which solutions would be developed. Owing to the complexity of sedentary behaviour, the identified barriers could be charted to nearly every domain of the TDF and COM-B instead of narrowing the intervention options as intended [57], a similar issue encountered by Hall et al. [39]. Consequently, the intervention components, including education [65], activity monitoring [66, 67], group sessions [68], goal setting and action planning [69], each target multiple determinants of sedentary [70, 71] and were tailored to the specific contexts of participants’ sedentary behaviour as highlighted during the behavioural diagnoses conducted in stage 1. Furthermore, integration of the COM-B within the BCW, helps consider broader socio-ecological influences on sedentary behaviour [70, 71].

Although the latter stages of the BCW translate to the co-production process, they were less congruent than stage one. A balance needed to be found between upskilling group members about current strategies and intervention components whilst not limiting creativity. To do so, group members generated solutions to barriers identified from the behavioural diagnosis before being presented with current strategies to reduce sedentary behaviour. The solutions selected by the group were then retrospectively coded to intervention functions and BCTs of the COM-B and BCW, respectively. As seen with other co-produced interventions [72–75], the selection of policy categories was less straightforward than in other BCW stages. Although no relevant policy categories were identified during the co-production process, the identification of policies can operate as an over-arching consideration rather than a distinct phase in the BCW process [73], and can also be identified during later stages of intervention development.

### General difficulties with the intervention development process

The co-production process outlined in this study involved significant time and effort from both the researchers and participants. While the approach followed was similar to those used by Hall et al.[39] and Wray et al.[72], the specific tasks and content were tailored to this study. Due to the iterative nature of the process, workshops required immediate analysis to inform the planning and content of subsequent workshops. Furthermore, workshop content had to map to the BCW framework whilst remaining engaging, accessible, and appropriate. Additionally, certain activities, such as mapping the behavioural diagnosis and intervention components to the BCW, TDF and BCT, were particularly time-consuming.

### Strengths and limitations

This study makes a valuable contribution to the existing body of literature on the development of complex interventions. Specifically, it outlines the utilisation of co-production and behaviour change theory principles in the process of intervention development. Transparency in reporting may help us understand which strategies lead to the development or refinement of effective interventions and help other researchers who are attempting to combine partnership and theory-based approaches to develop an intervention. Furthermore, the cumulative benefits from combining co-production and the BCW may result in interventions that are more feasible, acceptable, and effective than interventions developed using each strategy separately. This study is also important from an inclusion perspective, as older adults, particularly the oldest old, risk systemic exclusion from participatory research methods [76] due to being more likely to experience sensory and cognitive impairment [77]. This is evidenced by the comparatively slower adoption of co-production in this demographic compared to other groups [78–80]. This study demonstrates that co-production can be conducted with this sub-section of the population, but older adults may need additional assistance to effectively communicate their perspectives and requirements [78].

This intervention may have been limited by the recruited sample not being wholly representative of the wider population of community-dwelling older adults aged ≥ 75 years. Despite purposive sampling being employed with factors such as sedentary time, frailty status and living arrangements being considered, the recruited sample could not provide perspectives of older adults who are socially isolated or cultural perspectives on reducing sedentary behaviour in this population. In an attempt to mitigate this limitation, members of the co-production group were urged to consider older adults outside of the group members to ensure that the intervention would be appropriate for as many older adults as possible. Furthermore, additional perspectives from a preceding mixed-method systematic review were incorporated to provide wider perspectives on reducing sedentary behaviour in this population [52].

Despite trying to mitigate these limitations, all group members were able to travel to the workshops and share their views in a group setting. These prerequisites may have precluded many isolated older adults who are not comfortable leaving their homes or have unreliable public transport services from participating in the workshops, which are common barriers to reducing sedentary time [81]. As such, certain intervention components, such as group sessions, may be more acceptable or appropriate to older adults who are socially active than more socially-isolated members of the community. Semi-structured interviews with older adults who are socially isolated were considered to explore the acceptability or appropriateness of the developed intervention. However, these interviews would have provided insights into how they perceived the intervention components prior to their participation in the intervention. Instead, the acceptability of the intervention was explored in a feasibility study that will be reported elsewhere [53].

## Conclusions

This study successfully integrated co-production with the BCW to develop an intervention to reduce sedentary behaviour in community-dwelling older adults aged ≥ 75 years. The co-production process can enhance the feasibility of the developed intervention, whereas the BCW provided a structured framework to identify key intervention components, mechanisms of action and behaviour change techniques [41]. While the study addressed its main objectives, limitations remain, particularly the underrepresentation of socially isolated, and minority ethnic older adults. Although efforts were made to mitigate this, future research should explore alternative engagement methods, such as individual interviews or community-based outreach, to ensure inclusivity. Additionally, future studies should assess the intervention’s effectiveness in diverse populations. By systematically integrating theory and stakeholder perspectives, this study lays a foundation for future interventions and highlights the need for inclusive strategies to reduce sedentary behaviour in older adults.

## Supplementary Information

Supplementary Material 1.

Supplementary Material 2.

Supplementary Material 3.

## Data Availability

The data that support the findings of this study are available upon reasonable request from the corresponding author.

## Abbreviations

ASR: Ageing and Stroke Research
BCTs: Behaviour change techniques
BCWs: Behaviour change wheel
CARE75 +: Community Ageing Research 75 + Study
COM-B: Capability, opportunity, motivation-behaviour
eFI: Electronic Frailty Index
MOST: Measure of Older Adults' Sedentary Time
MRC: Medical Research Council
TDF: Theoretical domains framework
