# Supplementary figures and images for: Co-producing an intervention to reduce sedentary behaviour in community-dwelling older adults aged ≥ 75 informed by behaviour change theory
Source: BMC Geriatr. 2025 Mar 27;25:201. doi: 10.1186/s12877-025-05844-6 (PMC11951794; doi:10.1186/s12877-025-05844-6)

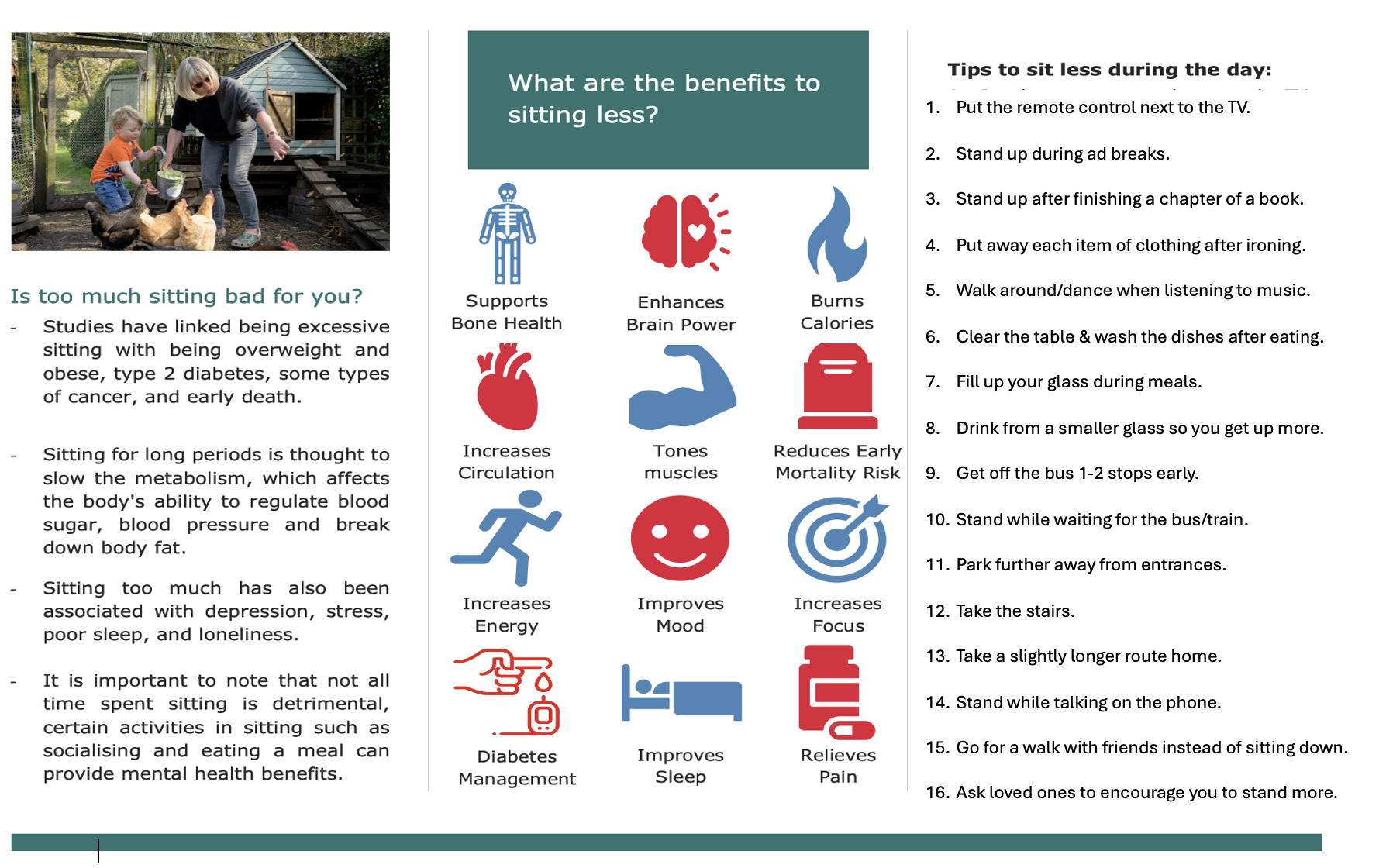


| **Week** | **Day 1** | **Day 2** | **Day 3** | **Day 4** | **Day 5** | **Day 6** | **Day 7** | **Comments** |
| --- | --- | --- | --- | --- | --- | --- | --- | --- |
| **1** |  |  |  |  |  |  |  |  |
| **2** |  |  |  |  |  |  |  |  |
| **3** |  |  |  |  |  |  |  |  |
| **4** |  |  |  |  |  |  |  |  |
| **5** |  |  |  |  |  |  |  |  |
| **6** |  |  |  |  |  |  |  |  |
| **7** |  |  |  |  |  |  |  |  |
| **8** |  |  |  |  |  |  |  |  |
| **9** |  |  |  |  |  |  |  |  |

Supplement: Supplementary file 1 — Supplementary Material 1. [file 12877_2025_5844_MOESM1_ESM.docx]
