# Supplementary material for: Co-producing an intervention to reduce sedentary behaviour in community-dwelling older adults aged ≥ 75 informed by behaviour change theory
Source: BMC Geriatr. 2025 Mar 27;25:201. doi: 10.1186/s12877-025-05844-6 (PMC11951794; doi:10.1186/s12877-025-05844-6)
Supplement: Supplementary file 2 — Supplementary Material 2. [file 12877_2025_5844_MOESM2_ESM.docx]

**Evaluation Form**

**To what extent do you agree with the following statements:**

1. **Transparency:** The overall aims, limitations, expectations and commitments of the project/groups were clearly explained to me.

| **Strongly disagree** | **Disagree** | **Neither agree**  **nor disagree** | **Agree** | **Strongly agree** |
| --- | --- | --- | --- | --- |
|  |  |  |  |  |

1. **Inclusivity:** The activities we performed were accessible and the group included a range of people that could capture individual and differing views.

| **Strongly disagree** | **Disagree** | **Neither agree**  **nor disagree** | **Agree** | **Strongly agree** |
| --- | --- | --- | --- | --- |
|  |  |  |  |  |

1. **Iterative:**  The content that we discussed was reciprocal, repeated, and progressive. Our discussions adapted and built upon what came before.

| **Strongly disagree** | **Disagree** | **Neither agree**  **nor disagree** | **Agree** | **Strongly agree** |
| --- | --- | --- | --- | --- |
|  |  |  |  |  |

1. **Positive:** The groups were beneficial and an overall positive experience. I felt valued, heard, engaged, and empowered. My expectations were met.

| **Strongly disagree** | **Disagree** | **Neither agree**  **nor disagree** | **Agree** | **Strongly agree** |
| --- | --- | --- | --- | --- |
|  |  |  |  |  |

1. **Equal:** I felt that my contributions and I were valued equally. Any problems and solutions discussed were tackled collaboratively. Any potential issues relating to power imbalances were addressed.

| **Strongly disagree** | **Disagree** | **Neither agree**  **nor disagree** | **Agree** | **Strongly agree** |
| --- | --- | --- | --- | --- |
|  |  |  |  |  |

1. **Impact:** I can understand the impact that these group meetings had/will have.

| **Strongly disagree** | **Disagree** | **Neither agree**  **nor disagree** | **Agree** | **Strongly agree** |
| --- | --- | --- | --- | --- |
|  |  |  |  |  |
