# Supplementary material for: Co-producing an intervention to reduce sedentary behaviour in community-dwelling older adults aged ≥ 75 informed by behaviour change theory
Source: BMC Geriatr. 2025 Mar 27;25:201. doi: 10.1186/s12877-025-05844-6 (PMC11951794; doi:10.1186/s12877-025-05844-6)
Supplement: Supplementary file 3 — Supplementary Material 3. [file 12877_2025_5844_MOESM3_ESM.docx]

Topic Guides for Qualitative Fieldwork

**Workshop 1 Interview Guide:**

- Introduction
- Rules
- Overview of session
- Video introduction

**Understanding of Sedentary Behaviour**

1. Had you heard the term “sedentary behaviour” before today, and if so could you describe it in your own words?
2. How would you describe your current levels of sedentary behaviour?
3. Are there any periods of day where you are more sedentary than others/less sedentary?
4. How has the amount you sit or lie down changed through the years? Has anything contributed to this?
5. How does sitting/lying down for long periods make you feel? Do you notice any effects?
6. How do you feel when you stand up? // What do you think the benefits of sitting less might be?

**Activities Performed in Sitting and Standing**

1. In front of you is a pen and notepad, I want everyone to take 5 minutes to write down as many activities and the reasons why you sit down throughout the day? – we’re going go around the group and make a big list of what we like to do.
2. We’re going to do a similar exercise, but we’re going to write down the things that the activities that are not in sitting.
3. How do you feel about incorporating more activity/moving around more into your day-to-day routine? **Have you taken up or tried to take up any new hobbies/activities?**
4. **Our final question deals with, we’re going to go around the table for this one, out of all the things we’ve talked about today, what to you is the most important thing that has been said – it might have been something you’ve said or something you heard someone else say - what do you think is most important – X can we start with you?**
5. **Did you have any final questions you wanted to ask this group – and if you don’t - would you give a little brief summary of the key points we talked about today**

**Recap**

1. **Did we capture that correctly? Is there anything that we missed or does that cover everything?**
2. **Dates – day and time preference – booking the room?**

| **COM-B Construct** | **COM-B Domain** | **TDF Domain** | Initial Question | Follow up/ Probing Questions |
| --- | --- | --- | --- | --- |
| **Capability** | **Psychological** | **Knowledge** | If a health care professional was to advise you to sit less, what do you think this might involve? | What do you think the benefits of sitting less might be? |
|  |  | **Memory, Attention, Decision Process** | What are the things that influence your decision to sit or stand? – follow up from the activities performed in sitting/standing | |
|  |  | **Behavioural Regulation** | Do you ever set any rules for yourself about when you should stand and move around? | |
|  | **Physical** | **Skills** | How easy or difficult would you find following that advice? | |
| **Opportunity** | **Social** | **Social Influences** | How do other people affect the amount of time you sit? | Anybody that helps or hinders reducing the time you sit? Perception of others? Groups? |
|  | **Physical** | **Environmental Context and Resources** | How does your environment affect the amount of time you sit? | Home – garden, stairs, space?  Neighbourhood – well maintained, safe, paths?  Travel – bus, train, car, cycle, walking?  Local facilities/clubs?  Time? Weather? |
| **Motivation** | **Reflective** | **Belief about Capabilities** | How confident are you that you would be able to reduce your sitting time?  How confident do you think these changes could be maintained?  What would help maintain these changes? | |
|  |  | **Belief about Consequences** | What might be the consequences of spending a lot of time sitting down?  How do you feel about these consequences?  What do you think will happen if you reduce the amount of time we sit? | |
|  |  | **Intention** | If an intervention was made, would you intend to follow it? | |
|  |  | **Goal** | To what extent would you follow it? | |
|  |  | **Optimism** | Do you think the barriers we’ve come up with today can be addressed/overcome? | |
|  | **Automatic** | **Emotion** | Does your sitting ever impact your mood/how you feel? | |
|  |  | **Reinforcement** | How would you incentivise sitting less during the day? | |

**Workshop 2 Interview Guide:**

**Meeting Guide Session 3**

1. **Presentation**

- Recap Session 1: Knowledge of sedentary behaviour, what we did in sitting and standing
- Recap Session 2: Barriers and facilitators present
- Overview of Session 3: Intervention components
- **Session 3 Introduction -** Todays workshop is going to focus on what the intervention might look.

I went through our discussions from the previous meetings and there were 5 main questions that jumped out at me that I thought I would get your thoughts on. - I thought we'd go through them one-by-one to get your thoughts and ideas on each one and see what we think about them. If you have any suggestions, please do feel free to share them – your say is very important. I’ll also share together the main established methods that we have available at our disposal and get your thoughts on them.

**Activity Monitoring**

- Seemed like from the first session, we don't really think about the amount of time we spend sitting until we were asked about it. **Would I be correct in saying that?**
- *Refer to PowerPoint – Display Jawbone + self-monitoring*
- This is an activity monitor, its worn around the wrist or around the waist and underneath clothes for generally a week at the start and end of an intervention.
- It's worn during all waking hours except during any activities such as bathing or showering etc.
- **You mentioned you had used one of them before in a study, would you mind telling us a little about how you found it? What did you like or dislike about it?**

1. **Device Prompts**

- These monitors can also be used as part of an intervention - these activity monitors can be used to deliver a gentle vibration if they detect that a person hasn't moved in some time - every 15, 30 60 minutes etc. Could I get your thoughts on that? How would you feel if these devices told you when to stand up?

1. **Self-Monitoring**

- The other option is self-monitoring - it's as the name suggests. It aims to get you to become more aware of when you have been sat for a long time. It can be done through tick sheets, or a pen and notebook. You can use it to take down how long are the periods you sit for or how many times you stand up during the day etc. Ultimately, it aims for you to recognise the time you spend sitting.
- Could I get your thoughts on this? **Of the two methods which one would you prefer? A device that tells you when you’ve been sitting too long, or monitoring it yourself?**

1. **Education**

- We’ve previously talked about education and what topics we would like to receive information on.
  - **What is sedentary behaviour? What are the consequences of sitting too much? What are the benefits of reducing the amount of time that we sat? How do we do so?**
  - Ideally, how would you like to receive this information? In-person, written, both?

1. **Prompting**

- We also talked about how we wanted an intervention that slotted around our lives, and that we wanted advice on how to reduce our sitting time. Put together some a set of tips on how to reduce our sitting time based on the activities we did in sitting. I was thinking of taking 5-10 minutes and discussing them with the person sitting beside you. Are there any ones you don't like? Are there any ones you like? Any that could be improved?

1. **Social Support**

- We talked about social aspect a lot during the first meeting and again in the second meeting.
- Would you like the intervention to include an element of social support?
- What would your thoughts be on including a group social element to an intervention be? Similar to a group such as this. // **What benefits do you think could be provided from this?**
- What would your thoughts be on being followed up with by a researcher during the intervention? // What would you like this follow up to include?
- Would you prefer a group element over researcher follow up? How would you feel about including both?

1. **Framing**

- If you were to sign up for an intervention, would you prefer the message to focus more on sitting less or standing more? Do these messages mean the same to you? Do you prefer one over the other?

1. **Recap**

- Co-moderator briefly goes through bullet points of main things discussed - did we capture that correctly? Anything you would like to add? Any questions co-moderator wants to pick up on/ask.

1. **Wrap up**

- I'm going to give you some homework.
- Notepad - keep track of the amount of time you spend sitting.
- Prompt sheet - with tips on how to incorporate more standing into your day
- I want you to try and do the things we've discussed today. I'll check in with you before the final session in December and see how you’re getting on with it.

**Meeting Guide Session 4**

1. **General Introduction**
2. **Presentation**

- Recap Session 1: Knowledge of sedentary behaviour, what we did in sitting and standing
- Recap Session 2: Barriers and facilitators present
- Recap of Session 3: Intervention components
- Overview of today’s session – Review of intervention components – took feedback on board.
- Evaluation of the workshops. Wrap up.

1. **Session 3 Introduction**

- Our final session today will focus on reviewing what the intervention might look like.
- I took all of your suggestions and feedback from the last session on board, so I thought today I would go through the different components, show you the changes I’ve made and get your thoughts on what the intervention might look like overall.

1. **Daily prompts**

- Wanted practical advice on how to reduce our sitting time based on the activities we performed in our daily activities.
- We did a useful activity where you let me know the tips that you liked/disliked and how we could improve the tips.
- Took your feedback on board when refining these tips to better suit this population.

1. **Becoming aware of our sitting time**

- There was a bit of debate about this one. Some members of the group preferred device-based prompts (watch delivers a reminder when you haven't moved after some time), others preferred self-monitoring so just taking down the amount of time sat in a day, and some preferred neither.
- **Devices** - have more detail about what these might look like - commercially available smartwatches - they will deliver a reminder if they detect you haven't moved >250 steps in the hour. The reminders would be delivered during a prespecified time period e.g. 8am - 7pm.
- **Self-monitoring: Could take down the number of steps they did by the end of the day.**
- How we have incorporated your feedback: We will be allowing participants to choose which method they would prefer. Both methods aim to accomplish the same thing - raise awareness of the time you spend sitting. Incorporating participant choice.

1. **Social Support**

- Consensus that we liked the thought of a group-based intervention, that would consist of 3-4 meetings.
- Would allow a researcher to deliver educational component, but also an opportunity to meet other people, share tips, advice and form new avenues for social support.
- Also felt that a researcher follow-up could tie in nicely with this social element – during the off weeks between sessions would receive a call from a researcher, see how you’re getting on with the programme, review/progressing goals set etc.

1. **Education**

- Topics we wanted: what is sedentary behaviour, the negative consequences of excessive sitting, benefits of reducing sitting time, and how to practically go about reducing our sitting time. Wanted this information both in person (potentially as part of the group sessions) and written (like the pamphlet).

1. **Framing:** wanted to focus more on sitting less – wanted to convey that sitting was not a harmless behaviour, but some emphasis on moving more. **How would you feel about doing exercises whilst seated?**

**What the Intervention Might Look Like**

- **Intervention itself is expected to last 9 weeks.**
- Assessment at baseline – thigh activity monitor
- Will be given list of tips to reduce sedentary behaviour and set up with the smart watch.
- 4 group sessions every two weeks – educational session, opportunity to meet people, share advice etc. Followed up in the week between sessions by researcher to review and progress goals that have been set. Assessment at end of intervention – thigh activity monitor



1. **What did we achieve during these workshops**

- **To get to this point – systematically review the published evidence, informed the content of these workshops.**

**Step 2 – where we are currently, collectively developed a solution - your input**

1. Developed better understanding of sedentary behaviour
2. Discussed options for supporting a reduction in sedentary behaviour in adults aged ≥75
3. Prototyped a solution
4. **What’s next?**

Testing the prototype solution in a small scale study in order to refine it.
